# Epigenetic regulation of PANoptosis: DNA methylation, histone modifications and non-coding RNAs

**DOI:** 10.17179/excli2025-9099

**Published:** 2026-01-20

**Authors:** Yogendra Singh, Muhammad Afzal, M. Arockia Babu, Surya Nath Pandey, Arcot Rekha, Gaurav Gupta, Imran Kazmi, Sami I. Alzarea, Omar Awad Alsaidan, Waleed Hassan Almalki, Salem Salman Almujri

**Affiliations:** 1 Department of Pharmacology, Maharishi Arvind College of Pharmacy, Ambabari, Jaipur, Rajasthan, India; 2 Department of Pharmaceutical Sciences, Pharmacy Program, Batterjee Medical College, Jeddah 21442, Saudi Arabia; 3 Department of Pharmacy, School of Medical and Allied Sciences, Galgotias University, Greater Noida, India; 4 Department of Pharmacology, Teerthanker Mahaveer College of Pharmacy, Teerthanker Mahaveer University, Moradabad 244001 Uttar Pradesh, India; 5 Dr. D.Y. Patil Medical College, Hospital and Research Centre, Pune 411018, Maharashtra, India; 6 Centre for Research Impact & Outcome, Chitkara College of Pharmacy, Chitkara University, Rajpura, Punjab, India; 7 Department of Biochemistry, Faculty of Sciences, King Abdulaziz University, Jeddah 21589, Saudi Arabia; 8 Department of Pharmacology, College of Pharmacy, Jouf University, Aljouf, Sakaka 72341, Saudi Arabia; 9 Department of Pharmaceutics, College of Pharmacy, Jouf University, Aljouf, Sakaka 72341, Saudi Arabia; 10 Department of Pharmacology and Toxicology, Faculty of Pharmacy, Umm Al-Qura University, Makkah, Saudi Arabia; 11 Department of Pharmacology, College of Pharmacy, King Khalid University, 61421 Asir-Abha, Saudi Arabia

**Keywords:** PANoptosis, epigenetic regulation, immunogenic cell death, cancer biomarkers, chromatin remodeling, non-coding RNAs

## Abstract

PANoptosome (Programmed Necrosis-Apoptosis Optosome) multiprotein complexes mediate the convergence of apoptosis, pyroptosis, and necroptosis. The ability of cells to undergo programmed inflammatory cell death is regulated by the epigenetic control of PANoptotic sensors, adaptors, and effectors, and has pivotal implications for their use in cancer therapies. DNA methylation suppresses the main PANoptotic pathways, such as RIPK3 (Receptor-Interacting Serine/Threonine-Protein Kinase 3), GSDME (Gasdermin E), and CASP8 (Caspase-8) that promote chemoresistance; hypomethylating DNA silencers resume PANoptotic sensitivity. BRD4 (Bromodomain-Containing Protein 4)/p300 (E1A-Associated Protein p300 - Histone Acetyltransferase) -mediated histone acetylation in enhancers (H3K27ac) stimulates ZBP1 (Z-DNA Binding Protein 1), NLRP3 (NOD-Like Receptor Family Pyrin Domain Containing 3), and caspase-8 transcription but inhibits the formation of inflammasomes by HDAC (Histone Deacetylase). PANoptotic regulatory regions become accessible in response to inflammatory signals through the dynamic regulation of accessibility through the SWI/SNF (Switch/Sucrose Non-Fermentable Chromatin Remodeling Complex) and NuRD (Nucleosome Remodeling Complex) and NuRD (Nucleosome Remodeling and Deacetylase Complex) chromatin remodelling complexes. Post-transcriptional regulation is mediated by ncRNAs (ncRNAs) such as miR-223-3p (MicroRNA-223-3p) and lncRNA NEAT1 (Long Non-Coding RNA - Nuclear Enriched Abundant Transcript 1) which converge to regulate the expression of NLRP3, RIPK3, and Gasdermin D (GSDMD). The interaction of DNA methylation, histone modification, and ncRNAs creates quantitative epigenetic thresholds that regulate PANoptotic sensitivity. The rational next step to overcome tumor immunoresistance is epigenetic biomarker stratification in combination with DNA methyltransferase inhibitors (DNMTi), histone deacetylase modulators (HDACi), and PANoptosis agonists, which could help reduce collateral tissue toxicity.

See also the graphical abstract(Fig. 1).

## Introduction

Programmed cell death (PCD) is necessary to maintain tissue homeostasis and immune defense (Lawlor et al., 2015[40]). Nevertheless, cancer cells and pathogens have used the plasticity of single death mechanisms, that is, apoptosis, pyroptosis, and necroptosis, to avoid immune clearance. PANoptosis, or Programmed Necrosis-Apoptosis, represents a significant shift from isolated pathway paradigms to an integrated cell death program (Pandeya and Kanneganti, 2024[56]; Tweedell et al., 2024[68]). In this process, PANoptosomes form complexes that activate the apoptotic, pyroptotic, and necroptotic pathways. This synchronized activation is necessary to provide potent inflammatory and immunogenic cell death, which is essential for antitumor immunity. Compared to monolithic cell death programs, PANoptosis offers functional redundancy by having multiple execution programs (Conos et al., 2017[13]; Xu et al., 2025[81]), and is therefore an attractive therapeutic target for overcoming immunoresistance in cancer and other diseases where normal cell death programs may be dysregulated.

The regulation of cellular competence to undergo PANoptosis is primarily governed by epigenetic mechanisms that control sensor stimulation, adaptor recruitment, and effector activation (Karki et al., 2021[32][33]). DNA methylation silences important PANoptotic pathways-RIPK3 (Receptor-Interacting Serine/Threonine-Protein Kinase 3), GSDME (Gasdermin E), and CASP8 (Caspase-8), leading to chemoresistance. However, PANoptotic sensitivity is regained by RIPK3 promoter repromethylation (Feng et al., 2025[17]; Koo et al., 2015[38]; Wang et al., 2024[70]). Transcriptional thresholds are established through histone modifications and chromatin remodeling, specifically via acetylation at enhancers (H3K27ac; Histone H3 Lysine 27 acetylation). This process is facilitated by CBP/p300 and recognized by BRD4 (Bromodomain-Containing Protein 4), involving the activation of PANoptotic sensors ZBP1 and NLRP3 and the deactivation of inflammasomes by Histone Deacetylase (HDAC) (Huang et al., 2020[26]; Tan et al., 2024[66]). PANoptotic regulatory regions can be dynamically regulated by accessibility dependent on SWI/SNF (Switch/Sucrose Non-Fermentable) and NuRD (Nucleosome Remodeling and Deacetylase) chromatin remodeling complexes in response to inflammatory signals (Baxter et al., 2023[4]; Wolf et al., 2024[79]). Rapid post-transcriptional control is provided by non-coding RNAs: miR-223-3p (MicroRNA-223-3p) and lncRNA NEAT1 (Long Non-Coding RNA - Nuclear Enriched Abundant Transcript 1) are regulators of NLRP3, RIPK3, and GSDMD (Gasdermin D) expression, which offers cell protection or sensitization to PANoptosis depending on the disease environment (Bennett et al., 2023[5]; Long et al., 2020[48]). These ncRNAs are associated with PANoptotic phenotypes and patient prognosis, making them useful biomarkers (Zhang et al., 2022[84]).

Interactions among DNA methylation, histone modifications, and non-coding RNAs produce quantitative epigenetic thresholds that mediate PANoptotic sensitivity and are quantifiable cell death competence parameters (Wang et al., 2023[73]). What is exceptional about such a mechanistic form of integration is that discrete epigenetic modifications map to particular architectural components of a PANoptosome, rendering epigenetic regulation a measurable trait that can be used therapeutically. Stratification of epigenetic biomarkers using DNA methyltransferase inhibitors (DNMTi), histone deacetylase modulators (HDACi), and PANoptosis agonist combination therapy is a logical solution to tumor immunoresistance with minimal collateral tissue toxicity (Guan et al., 2024[22]; Zhu et al., 2024[90]). This review synthesizes the three layers of epigenetic regulation: methylome, chromatin dynamics, and ncRNA networks, into a cohesive mechanism governing PANoptotic execution. This integration provides evidence-based insights into epigenetic-targeted cancer immunotherapy and precision oncology strategies for disorders characterized by dysregulated cell death.

## PANoptosis: Definitions, Triggers, and Core Machinery

PANoptosis (programmed necrosis-apoptosis) refers to a condensational cell death pathway that concurrently triggers apoptosis, pyroptosis, and necroptosis with the support of multiprotein PANoptosome complexes (Cai et al., 2023[8]; Wang et al., 2023[73]). Compared to isolated death pathway models, PANoptosis is a coordinated program that guarantees a good inflammatory program and immunogenic cell death (Taabazuing et al., 2017[65]). The PANoptosome combines sensors (ZBP1, AIM2, and NLRP3), adaptors (RIPK1/RIPK3, ASC, and FADD), and effectors (caspases-1/8, GSDMD/GSDME, and MLKL) to initiate several execution mechanisms in parallel (Christgen et al., 2020[11]). 

### PANoptosome sensors, adaptors, and effector assembly

Pathogenic or stress-generated signals trigger sensor activation. ZBP1 (Z-DNA Binding Protein 1) is a central PANoptosis initiator that senses endogenous RNA with the help of Z-alpha domains (Kuriakose et al., 2018[39]). NLRP3 (NOD-Like Receptor Family Pyrin Domain Containing 3) perceives danger-associated molecular patterns (DAMPs) (Wei et al., 2016[78]). AIM2 (Absent in Melanoma 2) is used to detect cytosolic double-stranded DNA (dsDNA) (Lee et al., 2013[42]). When these sensors are activated, adaptors RIPK1/RIPK3 (Receptor-Interacting Kinase 1/3), Apoptosis-Associated Speck-like Protein (ASC), and Fas-Associated Protein with Death Domain (FADD) are recruited to form PANoptosome scaffolds (Christgen et al., 2020[11]). These complexes interact with executioner caspases (caspase-1 and caspase-8) to activate GSDMD and D (Gasdermin) and gasdermin E (GSDME), creating membrane pores. Simultaneously, RIPK3 phosphorylates Mixed Lineage Kinase Domain-Like Protein (MLKL), which polymerizes at membranes and leads to necroptotic rupture (Conos et al., 2017[13]). The multiprotein complex exhibits functional redundancy, as the loss of individual effectors does not prevent the occurrence of PANoptosis (Lei et al., 2023[43]), and the inflammatory cell death process remains strong.

### Mitochondrial involvement in PANoptosis activation

Mitochondria are important amplification sites for PANoptosis. Cytochrome c is released by mitochondrial outer membrane permeabilization (MOMP) caused by the cleavage of BH3-Interacting Domain Death Agonist (BID), activating the apoptosome and caspase-9/caspase-3 cascade (Taabazuing et al., 2017[65]). GSDME is cleaved by caspase-3, which transforms apoptotic pores into pyroptotic pores. Reactive oxygen species (ROS) production is also induced by mitochondrial stress, which increases the effect of inflammasome activation and augments PANoptotic signals (Orning et al., 2018[55]; Wu et al., 2025[80]). Gasdermin pores cause calcium flux, leading to further mitochondrial impairment, forming positive feedback loops that lead to total cell elimination. Epigenetic regulation also influences mitochondrial checkpoints, with DNA methylation of genes that promote cell death and histone modifications affecting the expression of mitochondrial proteins, thereby determining the PANoptotic capability (He et al., 2020[24]; Stolz et al., 2022[64]). 

### Signal transduction crosstalk among apoptosis, pyroptosis, and necroptosis

PANoptosis involves interactions between pathways rather than their independent activation. RIPK3 serves as a central point for both necroptosis (via MLKL) and pyroptosis (through the activation of the NLRP3 inflammasome without MLKL involvement) (Bozgeyik et al., 2024[6]; Lawlor et al., 2024[41]). Activated MLKL promotes the assembly of the NLRP3 inflammasome, which constitutes a necroptosis-to-pyroptosis cascade (Mobley et al., 2017[52]). Conversely, under normal conditions, caspase-3 cleaves gasdermin D to inhibit inflammasome-mediated pyroptosis and stimulate apoptosis (Taabazuing et al., 2017[65]). Nonetheless, extrinsic apoptosis (death receptor signaling) is linked to pyroptotic pore formation via the cleavage of GSDMD/GSDME by caspase-8, especially in bacterial infections or in response to TNF-α (Sarhan et al., 2018[62]). Synergism with TNF-α and IFN-γ stimulates the expression of the transcriptional factors STAT1 and IRF1 (Interferon Regulatory Factor 1), resulting in the upregulation of ZBP1, caspase-8, RIPK3, and GSDMD, which form the conditions that favor PANoptotic execution (Deng and Hu, 2023[15]; Kim et al., 2023[35]). Blocking any single effector pathway does not stop cell death, highlighting the mechanistic redundancy characteristic of PANoptosis, which ensures that the pathogen is eliminated even when specific pathways are inhibited (Lei et al., 2023[43]). 

### Disease relevance and biomarker potential

Dysregulation of PANoptosis contributes to the pathology of cancer, infection, and autoimmune conditions (Devos et al., 2020[16]). Chemoresistance in tumors is formed by epigenetic silencing of PANoptotic effectors (, especially RIPK3 and GSDME, via promoter hypermethylation) (Arroyo Villora et al., 2024[1]). In cases of inflammation, excessive PANoptosis triggered by DNA damage that activates ZBP1 leads to tissue toxicity (Wang et al., 2024[71]). The patterns of GSDME methylation and CASP8 expression are biomarkers of treatment response (Martinez et al., 2007[50]), and miR-223-3p (MicroRNA-223-3p) regulates both NLRP3-dependent pyroptosis and RIPK3-dependent necroptosis (Bennett et al., 2023[5]; Long et al., 2020[48]), which is a post-transcriptional regulatory node controlling PANoptotic thresholds. The epigenetic status of PANoptotic components, especially the RIPK3, GSDME, and CASP8 patterns of methylation, allows patient stratification for the personal selection of epigenetic priming agents in epigenetic splits versus PANoptosis activators (Koo et al., 2015[38]). Epigenetic reactivation of silenced PANoptotic effectors by DNA methyltransferase inhibitors (DNMTi) and histone deacetylase inhibitors (HDACi) restores PANoptotic competence, defining epigenetic regulation as an important determinant of therapeutic efficiency (Gong et al., 2023[20]).

## Epigenetic Mechanisms Relevant to PANoptosis

Epigenetic mechanisms, consisting of three interconnected layers-DNA methylation, histone modifications/chromatin remodeling, and non-coding RNAs-regulate a cell's ability to undergo PANoptosis. These pathways act as measurable indicators that determine whether a cell will proceed with PANoptosis or evade cell death (Pandeya and Kanneganti, 2024[56]; Tweedell et al., 2024[68]).

### DNA methylation: writers, erasers, and readers in PANoptosis control

PANoptotic sensor and effector expression is regulated by DNA methylation regulators: writers (DNMT), erasers (TET), and readers (UHRF1/MBDs) (Chiappinelli et al., 2015[10]). Inhibition of DNA Methyltransferase (DNMT) restores endogenous retroviral elements (ERVs) to produce cytosolic double-stranded RNA (dsRNA) during endogenous stimulation of type I interferon signaling in preconditioning PANoptotic death (Wei et al., 2016[78]). In contrast, hypermethylation of PANoptotic effectors mediated by DNMT, such as RIPK3, GSDME, and CASP8, inhibits necroptosis and pyroptosis, leading to chemoresistance in cancer (Koo et al., 2015[38]). These loci are demethylated through the action of Ten-Eleven Translocation (TET) enzymes, which reinstates PANoptotic potential (Stolz et al., 2022[64]). TET1 is also epigenetically independent of ERVs repression by chromatin remodeling, limiting ZBP1 activation. One of the methyl-CpG readers is UHRF1 (Ubiquitin-Like PHD and RING Finger Domain Containing Protein 1), which identifies H3K9me3 patterns at retroviral sequences and inhibits aberrant innate immune activation (Irwin et al., 2023[29]). Combined ERV-stimulated DNMT and TET inhibition promote ERV-derived dsRNA to augment interferon reactions and immunotherapies (Huang et al., 2024[27]). Antitumor mechanisms can work synergistically with the loss of UHRF1 checkpoint inhibition, implying epigenetic reader-targeted therapy (Tan et al., 2024[66]). In clinical therapy, 5-azacitidine (a DNMT inhibitor) paired with anti-HER2 therapy increases the immunogenicity of ZBP1, which validates epigenetic-therapeutic combinations (Lin et al., 2020[44]). Further research will involve locus-specific mapping of methylation patterns at PANoptosis-regulating loci and TET-dependent enhancer circuits to develop rational combination therapies.

### Histone modifications and chromatin remodeling: establishing transcriptional thresholds

Histone acetylation/deacetylation and ATP-dependent chromatin remodeling establish epigenesis that controls the expression of epigenetic PANoptotic genes (Raisner et al., 2018[59]). CBP/p300 (CREB-Binding Protein/p300) acetyltransferases mediate the H3K27ac (Histone H3 Lysine 27 acetylation) mark of active enhancers of PANoptotic sensors (ZBP1, NLRP3) and effectors (CASP8, GSDMD). BRD4 (Bromodomain-Containing Protein 4) is known to recognize acetylated histones and promotes transcription elongation at such loci (Kuriakose et al., 2018[39]). In contrast, deacetylation via Histone Deacetylase (HDAC) represses inflammasome activation. NLRP3 and GSDMD expression is potentiated by the inhibition of HDAC2 (Guan et al., 2024[22]), exposing cancer cells to PANoptosis. SWI/SNF and Nucleosome Remodeling and Deacetylase (NuRD) complexes are dynamically regulated to regulate PANoptotic regulatory region accessibility in response to inflammatory signals by ATP-dependent nucleosome repositioning (Gresh et al., 2005[21]). SWI/SNF subunits induce programs of inflammatory repression by NuRD and programs of lineage-specific death by SWI/SNF subunits (Baxter et al., 2023[4]; Wolf et al., 2024[79]). Chromatin remodeling to re-express RIPK3 restores necroptotic signaling in recurrent breast cancer, making the chromatin state a competency regulator (Lin et al., 2020[44]). PANoptosis is also controlled by histone methylation: H3K27me3 (repressive mark) deposited by PRC2/EZH2 represses NLRP3, and silencing EZH2 by blocking viral mimicry leads to ZBP1 activation (Deng and Hu, 2023[15]; Kim et al., 2023[35]). Conversely, H3K9me3 settles due to SETDB1 inhibitors necroptosis mediated by ZBP1 silencing of retroelements (Zheng and Kanneganti, 2020[86]). H3K27me3 is demethylated by JMJD3 (KDM6B) to activate NLRP3 transcription and pyroptosis (Huang et al., 2020[26]). These coordinated modifications characterize epigenetic changes, where repressive shifts or the inhibition of BRD4/acetyltransferase render cells susceptible to PANoptosis (Martire et al., 2019[51]). Future research should aim to identify locus-specific histone patterns and specific epigenetic regulatory agents that influence PANoptosis for clinical use.

### Non-coding RNAs: post-transcriptional regulation and biomarker potential

Non-coding RNAs (miRNAs, lncRNAs, circRNAs) regulate PANoptotic activation thresholds by regulating the expression of CASP8, RIPK3/MLKL, and NLRP3 and act as post-transcriptional inhibitors of PANoptosis (Zhang et al., 2019[83]). miR-223-3p (MicroRNA-223-3p) antagonistically regulates NLRP3-dependent pyroptosis and RIPK3-dependent necroptosis (Bennett et al., 2023[5]; Long et al., 2020[48]). High levels of miR-223 are associated with inflammatory diseases; miR-223 replacement therapy can restore PE-regulated pyroptotic/necroptotic thresholds (Jia et al., 2024[30]). miRNA NEAT1 (Nuclear Enriched Abundant Transcript 1) is a nuclear scaffold that organizes PTBP1-FOXP1 regulatory cascades to induce pyroptosis by NLRP3 and forms inflammasomes by organizing its paraspeckle (Liu et al., 2023[47]; Zhou et al., 2024[89]). The expression of NEAT1 is associated with PANoptotic signatures and prognosis; therefore, circNEIL3 can be used as a biomarker. circNEIL3 is an endogenous competitor of miR-1184, which reduces PIF1 and increases AIM2-mediated pyroptosis (Zhang et al., 2019[83]). Intercellular PANoptotic signaling- exosomal ncRNAs transporting circHIPK3 or miR-378 suppress NLRP3 pyroptosis, which has therapeutic potential. MiR-223 replacement, NEAT1 destabilization in epithelial disorders, and circRNA hub targets are strategies that can be employed to achieve specific outcomes. These regulatory mechanisms are integrated into measurable biomarker panels that predict therapeutic responses.

## DNA Methylation Landscapes Shaping PANoptosis

DNA methylation at the loci of PANoptotic components regulates the cellular capacity for programmed cell death and serves as a key biomarker for cancer therapies (Koo et al., 2015[38]). The hypermethylation of locus-specific PANoptotic sensors (NLRP3, ZBP1, AIM2), adaptors (ASC/PYCARD, RIPK1/RIPK3, FADD), and executors (CASP8, GSDME, GSDMD, MLKL) is therapeutically reversible (Hubert and Rich, 2022[28]).

### Methylation states at PANoptotic components in cancer

Chemoresistance in various tumors is mediated by epigenetic silencing of PANoptotic effectors via promoter hypermethylation. hypermethylation of the RIPK3 (Receptor-Interacting Serine/Threonine-Protein Kinase 3) is one of the hallmarks of necroptosis inhibition. In non-small cell lung cancer (NSCLC), RIPK3 hypermethylation is associated with cisplatin resistance (Ohashi et al. 2019[54]). RIPK3 hypermethylation restores RIPK3 expression and chemosensitivity in xenografts (Zhou et al., 2022[87]). This trend is generalized to solid tumors RIPK3 silencing through hypermethylation, a universalized process, and inhibition of necroptotic pathways in melanoma, osteosarcoma, and recurrent breast cancer (Arroyo Villora et al., 2024[1]; Zhou et al., 2024[88]). Inflammatory cell death induced by chemotherapy is inhibited by hypermethylation of the GSDME/DFNA5 (Gasdermin E/Deafness Autosomal Recessive 5) promoter, which blocks pyroptotic execution in colon and breast cancers (Vaira et al., 2007[69]). Hypermethylation of the CASP8 (Caspase-8) CpG-islands in CASP8 in relapsed glioblastoma is an adaptation specific to relapse (Martinez et al., 2007[50]), which inhibits apoptosis. ASC/PYCARD (apoptosis-associated speck-like protein containing a CARD / Pyrin and CARD domain containing) hypermethylation in prostate cancer is linked to immune sensor-mediated caspase-1 inhibition and decreased pyroptotic ability (Collard et al., 2006[12]). The inhibition of gastric cancer by pyroptotic execution due to GSDMD (Gasdermin D) promoter hypermethylation is correlated with malignant progression (Muhammad et al., 2019[53]). In contrast, hypomethylation-based patterns in leukemias point to tissue-specific plasticity: low NLRP3 (NOD-Like Receptor Family Pyrin Domain Containing 3) and CASP1 (Caspase-1) promoter methylation is associated with high levels of expression and glucocorticoid resistance, which suggests that PANoptotic responses are dynamically controlled by methylation (Wei et al., 2016[78]). These circumstance-dependent locus-specific methylation states are regulators of sensor-adaptor-executor modules controlling circumstance-dependent formation of tissues and disease resistance to therapy (Table 1(Tab. 1); References in Table 1: Baik et al., 2021[2]; Collard et al., 2006[12]; Deng and Hu, 2023[15]; Kim et al., 2023[35]; Koch et al., 2021[36]; Konno et al., 2018[37]; Koo et al., 2015[38]; Martinez et al., 2007[50]; Muhammad et al., 2019[53]; Pandeya and Kanneganti, 2024[56]; Paugh et al., 2015[57]; Wei et al., 2016[78]; Zhou et al., 2022[87]).

### Interferon and inflammatory gene program regulation by DNA methylation

The principal mechanism of type I interferon (IFN) and inflammatory signaling via DNA methylation determines PANoptotic priming. Endogenous retroelements are deregulated by DNMT inhibition (5-azacitidine or decitabine) to produce cytosolic double-stranded RNA (dsRNA) that activates MDA5/MAVS (Melanoma Differentiation-Associated Protein 5 / Mitochondrial Antiviral Signaling Protein), which results in type I IFN production by IRF7 and upregulation of interferon-stimulated genes (ISGs) (Chiappinelli et al., 2015[10]; Roulois et al., 2015[61]). This mimicry of viral reactions enhances the PANoptotic ability in colorectal and ovarian cancer models. The cytosolic DNA-sensing (cyclic GMP-AMP synthase (cGAS) - stimulator of interferon genes (STING) pathway is another important target of DNA methylation. The promoter of cGAS-STING is silenced by cytosine methylation in tumor cells (Konno et al., 2018[37]).

Pharmacological demethylation of this pathway reinstates the cGAS-STING-mediated innate immune response and suggests that epigenetic methylation is a regulatory node that dictates PANoptotic outcomes. TNF-alpha and IFN-sigma (Tumor Necrosis Factor-Alpha and Interferon-Gamma) synergy leads to the transcriptional activation of PANoptotic executors (CASP8, GSDMD, RIPK3, and MLKL) by IRF1 and STAT1 (Signal Transducer and Activator of Transcription 1) transcription factors. Inhibition of this regulated inflammatory cell death is not observed upon loss of any one of these effector pathways, demonstrating PANoptotic mechanistic redundancy (Deng et al., 2024[14]; Karki et al., 2021[33]). One of the interferon-stimulated genes that have been shown to be essential in PANoptosome scaffolding (Cai et al., 2023[8]; Wang et al., 2023[73]), ZBP1 (Z-DNA Binding Protein 1) is silenced by epigenetic promoter hypermethylation at locus cg09897064, reducing CEBPA (CCAAT/Enhancer Binding Protein Alpha) binding to the promoter. This epigenetic modification changes macrophage polarization to immunosuppressive M2 macrophages and predetermines poor outcomes in lung adenocarcinoma (Low et al., 2022[49]). STING repression is a reversible program of development in gliomas, which can be reversed by methyltransferase inhibition, indicating that hypomethylating therapy can sensitize tumors to PANoptotic strategies.

### Therapeutic reversibility and biomarker-guided clinical translation

Hypermethylation-induced PANoptotic silencing can be reversed to reverse chemotherapy resistance by 5-azacitidine (5-aza-dC) to restore GSDME expression and pyroptotic activity in breast cancer models (Vaira et al., 2007[69]). Decitabine demethylates GSDME in breast cancer demethylates GSDME and enhancing pyroptosis and chemosensitivity. In isocitrate dehydrogenase-mutant acute myeloid leukemia (Isocitrate Dehydrogenase-mutant AML), decitabine reinstates RIPK3-expression and overcomes necroptotic suppression in metabolic dysregulation (Gong et al., 2023[20]). Guadecitabine (SGI-110) is a long-acting hypomethylating agent that reverses Martin Lewis Kindred Lymphoma (MLKL) hypermethylation in Burkitt lymphoma (Koch et al., 2021[36]), and reinstates necroptosis with TNF and second mitochondria-derived activator of caspase/TNF-related apoptosis-inducing ligand (SMAC/TRAIL) (Hubert and Rich, 2022[28]). An example of cGAS-STING axis reversibility is seen in gliomas, where decitabine administration reinstates immune sensing. Epigenetic demethylation in combination with conventional cytotoxics is more effective; for example, 5-azacitidine in combination with anti-HER2 therapy stimulates ZBP1 immunogenicity in tumors. These clinical cases define the status of methylation as a measurable biomarker for therapeutic choice (Baik et al., 2021[2]). The stratification of patients to undergo hypomethylating agent priming before the administration of PANoptosis agonists through biomarker-based stratification using RIPK3, GSDME, CASP8, and ZBP1 methylation presents an opportunity to select patients with these methylation patterns (Zhou et al., 2022[87]). A combination of high-resolution CpG methylation profiling and pharmacodynamic measurements of re-expressed PANoptotic proteins must become routine in future clinical trials (Arroyo Villora et al., 2024[1]; Zhou et al., 2024[88]). Future studies should also establish patterns of global methylation in the PANoptotic pathway (ZBP1-ASC-CASP1-CASP8-RIPK3-MLKL-GSDMD/GSDME) to improve the predictive analysis of PANoptotic responses to direct individualized cancer therapy and allow precision oncology in immunoresistant tumors.

## Histone Modifications and Chromatin Accessibility in PANoptosis

Quantifiable epigenetic thresholds that regulate PANoptotic gene expression are established via histone acetylation/deacetylation and ATP-dependent chromatin remodeling (Raisner et al., 2018[59]). These dynamic changes govern the transcription of PANoptotic sensors (ZBP1, NLRP3), adaptors (RIPK1/RIPK3, ASC), and executors (CASP8, GSDMD, GSDME, MLKL), which in turn determine cell PANoptotic competence and response to cancer therapy (Kuriakose et al., 2018[39]).

### Acetylation and histone deacetylase inhibition in PANoptotic sensitization

Active enhancers are marked by histone acetylation of H3K27ac (histone acetylation) catalyzed by CBP/p300 (CREB-Binding Protein p300 E1A-Associated Protein) histone acetyltransferases (HATs) at PANoptotic loci (Martire et al., 2019[51]). BRD4 (Bromodomain-Containing Protein 4) is a histone acetylation recognition protein at such enhancers, which enhances the transcriptional elongation of PANoptotic sensors and effectors (Bressin et al., 2023[7]). High-resolution nascent RNA analyses have confirmed that the induction of enhancers requires the presence of BRD4 and its interaction with enhancer-promoters, which in turn stimulates cytokines, interferon-stimulated genes, and the assembly of caspases into the PANoptosome (Wang et al., 2017[75]). The main transcriptional regulator IRF1 (Interferon Regulatory Factor 1) has been shown to activate ZBP1, AIM2 (Absent in Melanoma 2), RIPK1, and NLRP12, the combination of which in myeloid cells integrates signals of inflammatory and programmed cell death (Sharma et al., 2025[63]). Deacetylation mediated by histone deacetylase (HDAC), on the other hand, inhibits the release of inflammasomes and the execution of PANoptosis (Gagliano et al., 2024[18]; Zamperla et al., 2024[82]). Deacetylation of NLRP3 and GSDMD promoters by HDAC2 suppresses colorectal carcinoma; HDAC2 pharmacological or genetic inhibition leads to upregulation of these genes, which exposes cancer cells to PANoptosis (Guan et al., 2024[22]). Genome-wide H3K27ac levels are determined by the dynamic balance between CBP/p300 acetyltransferase and HDAC deacetylase activities, which create a transcriptional rheostat that is reversible between stress-responsive and death-associated genes (Wang et al., 2022[74]). BD Raf4 mediates NLRP3 (NOD-Like Receptor Family Pyrin Domain Containing 3) inflammasome and GSDMD-mediated hepatocyte pyroptosis in metabolic inflammation, and Bromodomain and Extra-Terminal (BET) bromodomain inhibition prevents this PANoptotic pathway, proving that the acetylation-dependent transcriptional regulation of cell death execution is vital (Chen et al., 2024[9]). The effects of HDAC inhibitors (HDACi) on PANoptotic sensitization involve three processes: augmentation of H3K27ac at PANoptotic regulatory sites, BRD4 recruitment, and inflammatory transcriptional programs (Gagliano et al., 2024[18]; Zamperla et al., 2024[82]). These These results This study places the HAT/HDAC equilibrium as an epigenetic parameter that can be quantified to regulate PANoptotic competence.

### Chromatin remodeling complexes and enhancer accessibility

Dynamic regulation of PANoptotic regulatory regions involving ATP-dependent chromatin remodeling complexes, Switch/Sucrose Non-Fermentable (SWI/SNF) and NuRD (Nucleoside Remodeling and Deacetylase), in response to inflammatory and pathogenic signals is subsequently regulated by dynamic remodelling of their accessibility (Gresh et al., 2005[21]). SWI/SNF subunits cause lineage-specific gene programs that organize the molecular architecture to initiate programmed cell death pathways (Baxter et al., 2023[4]; Wolf et al., 2024[79]). SWI/SNF-mediated chromatin remodeling and epigenetic re-expression of RIPK3 (Receptor-Interacting Serine/Threonine-Protein Kinase 3) restores necroptotic signalling in recurrent breast cancer, making the chromatin state a predictor of necroptotic competency and PANoptotic integration (Lin et al., 2020[44]). NuRD complexes can suppress the expression of inflammatory genes, maintaining a homeostatic state (Baxter et al., 2023[4]; Wolf et al., 2024[79]). ZBP1-RIPK3-caspase-8-GSDMD signaling axis requires much reliance on the states of chromatin accessibility and promoter repression controlled by these remodeling complexes (Lin et al., 2025[45]). NINJ1 (Ninjurin-1), which is responsible for the upstream activation of acetylation enhancers, mediates infection -and stress-induced PANoptosis, causing plasma membrane rupture (Han et al., 2024[23]). The ZBP1-NINJ1 axis is regulated by the interferon regulatory factor and regulates liver cell PANoptosis, a complex process involving coordinated pathogen sensing, activating enhancers, and rupturing terminal membranes (Qin et al., 2025[58]). Future studies should define locus-specific histone modification patterns and situation-specific chromatin remodeling to guide the development of selective epigenetic modulators for use in PANoptosis in clinical settings.

### Histone methylation in repressing and activating PANoptotic pathways

Histone methylation controls PANoptotic-related gene transcription through writers, erasers, and reader proteins (Deng and Hu, 2023[15]; Kim et al., 2023[35]). H3K27me3 (Histone H3 Lysine 27 Trimethylation) (repressive mark) deposited by PRC2/EZH2 (Polycomb Repressive Complex 2 / Enhancer of Zeste Homolog 2) suppresses NLRP3 transcription (Kim et al., 2008[34]). Repetitive elements are activated by EZH2 inhibition through viral mimicry pathways that activate ZBP1 and induce PANoptosis (Deng and Hu, 2023[15]; Kim et al., 2023[35]). This NLRP3 transcriptional activation and inflammasome-mediated pyroptosis are a result of JMJD3 (KDM6B; Lysine Demethylase 6B), a histone demethylase that removes H3K27me3 marks (Huang et al., 2020[26]). In contrast, the repressive mark (H3K9me3) (Histone H3 Lysine Methyltransferase 1) deposited by SETDB1 (SET Domain Bifurcated Histone Lysine Methyltransferase 1) silences endogenous retroviruses and prevents ZBP1-mediated necroptosis by protective chromatin structures (Zheng and Kanneganti, 2020[86]). SETDB1 inhibits the activation of endogenous retrosomes and ZBP1-mediated necroptosis via H3K9 methylation. These linked histone methylation and demethylation processes through PRC2/SETDB1 and KDM6B, along with reception by BET reader proteins and the interaction of ZBP1 with ZBP1-RIPK3-caspase-8-NLRP3, establish epigenetic prerequisites permitting ZBP1-RIPK3-caspase-8-NLRP3 axis expression (Kanno et al., 2014[31]). Chromatin repression or inhibition of BET proteins makes cells susceptible or resistant to PANoptosis. To translate this clinical information, it is necessary to create selective epigenetic modulators that can selectively activate PANoptotic pathways that mediate a balance between therapeutic and immunopathological activation. Mechanistic models that combine HAT/HDAC acetylation homeostasis with histone methylation conditions and chromatin remodeling complex action form the basis of multi-targeted epigenetic therapeutics in cancer immunotherapy (Baxter et al., 2023[4]; Wolf et al., 2024[79]).

## Non-Coding RNAs Regulatory Networks in PANoptosis

Non-coding RNAs (ncRNAs), such as microRNAs (miRNAs), long non-coding RNAs (lncRNAs), and circular RNAs (circRNAs), establish post-transcriptional regulatory interactions that modulate PANoptotic activation thresholds (Gong et al., 2024[19]; Pandeya and Kanneganti, 2024[56]). These ncRNA-mediated mechanisms enable cells to respond to both pathogenic and therapeutic stimuli. The core PANoptotic effectors CASP8, RIPK3/MLKL, and NLRP3 are regulated by the ncRNA signature, which serves as a reliable biomarker for PANoptotic phenotypes and therapeutic responses (Bennett et al., 2023[5]; Long et al., 2020[48]).

### MicroRNAs as post-transcriptional brakes and switches in PANoptosis

MicroRNAs balance PANoptotic pathways by convergent inhibition of death effectors. miR-223-3p (MicroRNA-223-3p) is a regulatory hub that convergently inhibits NLRP3-dependent pyroptosis and RIPK3-dependent necroptosis. miR-223-3p is a post-transcriptional brake on PANoptotic execution (Bennett et al., 2023[5]; Long et al., 2020[48]). High concentrations of miR-223 are associated with inflammatory diseases, and the restoration of normal levels of miR-223 corrects abnormal pyroptotic and necroptotic thresholds (Jia et al., 2024[30]). In contrast, miR-874 (MicroRNA-874) directly suppresses the expression of CASP8 (Caspase-8) to suppress apoptotic signals and reorganize cell death programs to induce necroptosis and assemble miRNA switches of PANoptotic fate choices (Wang et al., 2024[70]). These control systems generate a continuum of miRNA-mediated cell-death points. miR-223 dosage is an anticipatory factor for the inflammatory set-point and apoptotic equilibrium in tissues (Bennett et al., 2023[5]; Long et al., 2020[48]). Intercellular PANoptotic communication occurs in macrophages, in which miR223-3p is released through exosomes to inhibit necroptosis in severe atherosclerosis. Subcategorization of cancer based on PANoptotic potential and treatment response Disease-specific miRNA signatures, such as miR-18a (MicroRNA-18a) which suppresses HIF1a-NLRP3 under osteogenic conditions can be used (Zhang et al., 2022[84]). Future research should measure miRNA signatures at PANoptotic loci to stratify patients and forecast therapeutic responses.

### Long non-coding RNAs operating as nuclear scaffolds and regulatory hubs

Long non-coding RNAs serve as architectural regulators of inflammasome assembly and PANoptotic signaling dynamics signatures (Bao et al., 2024[3]; Hong et al., 2025[25]; Wang et al., 2024[72]). lncRNA NEAT1 functions as a regulatory factor of the innate immune system: it organizes the PTBP1-FOXP1 (Polypyrimidine Tract Binding Protein 1 - Forkhead Box Protein P1) regulatory cascades that drive NLRP3-induced PANoptotic phenotypic responses, and patient responses are associated with NEAT1 expression, which makes NEAT1 a dual-function biomarker and a regulator that allows subclassification of cancers based on PANoptotic competence (Liu et al., 2024[46]). Nuclear organization mediated by NEAT1 changes inflammasome effects in disease conditions with significant apoptotic implications. lncRNA panels can stratify cancers based on their potential to induce cell death (Bao et al., 2024[3]; Hong et al., 2025[25]; Wang et al., 2024[72]). NEAT1 destabilization is a therapeutic intervention for epithelial pathologies to overcome inflammasome-mediated pyroptosis in pathological models (Liu et al., 2024[46]). The results indicate that lncRNA scaffolding is a therapeutically targetable and measurable process by which the execution of PANoptotics is regulated by post-transcriptional RNA organization.

### Circular RNAs as competing endogenous RNAs modulating PANoptotic signaling

Circular RNAs are also competing endogenous RNAs (ceRNAs), where miR-NEIL3 (MicroRNA-1184) is bound by circNEIL3 (Circular RNA of NEIL3 Gene), leading to the de-repression of PIF1 (PAF1C-Associated Factor 1) (Zhang et al., 2022[85]). The circNEIL3-miR-1184-PIF1 axis facilitates DNA damage signaling and AIM2 (Absent in Melanoma 2) pyroptosis and correlates genotoxic stress-induced inflammasome responses with inflammasome signaling and the control of myeloid inflammatory cell death and apoptotic crosstalk in vivo. circRNAs promote pyroptosis by alveolar macrophages in innate immune cells during acute lung injury (ALI). CircHIPK3 (Circular RNA from HIPK3 Gene) and miR-378 (MicroRNA-378) exosomal circRNAs mediate intercellular PANoptotic signaling. NLRP3 pyroptosis is inhibited in recipient cells by exosomal circHIPK3 (Circular RNA from HIPK3 Gene) or miR-378 (MicroRNA-378) exosomal circRNAs, and the therapeutic potential of exosomal nc CeRNA hubs are quantifiable biomarkers and therapeutic targets in PANoptosis-related pathology through these circRNA regulatory mechanisms (Pandeya and Kanneganti, 2024[56]).

### Integrated ncRNA networks as predictive biomarkers and therapeutic targets

ncRNAs are collectively involved in the regulation of PANoptotic activation thresholds through the regulation of CASP8, RIPK3/MLKL, and NLRP3 at various regulatory nodes via quantifiable biomarker signatures of therapeutic responsiveness (Wang et al., 2023[77]). The selectivity between ncRNA signatures of activators and inhibitors of PANoptosis is possible through patient stratification (Liu et al., 2024[46]). Combinations of ncRNA-effector interactions provide superior PANoptotic control: pyroptotic/necroptotic signatures are re-established with miR-223 replacement, nucleation complexes are destabilized with NEAT1 inhibition, and circRNA hub targeting overcomes the effects of genotoxic stress. Mechanism enhanced by high-resolution ncRNA-effector interaction mapping of PANoptosis loci. The kinetic characterization of ceRNA-ceRNA competition during cell stress conditions involves the quantitative modeling of the competitive process by ncRNAs and the recruitment of pharmacological PANoptosis modulators with ncRNA targeting, which is the future of precision immunotherapy in immunoresistant cancer (Wang et al., 2023[77]).

## Integrating Epigenetic Regulation into PANoptosis-Based Cancer Immunotherapy

This review determines epigenetic regulation as a central determinant of cellular PANoptotic competence, which has far-reaching implications for cancer therapeutics. This interaction between DNA methylation, histone modifications, and non-coding RNA networks forms measurable epigenetic-based thresholds governing the execution of programmed inflammatory cell death versus the escape of immunogenic cell death (Chiappinelli et al., 2015[10]; Roulois et al., 2015[61]). This epigenetic paradigm has transformed PANoptosis from a mechanistic curiosity into a workable platform for precision oncology.

### Integrated epigenetic model of PANoptotic competence

The complexity of epigenetic regulation of PANoptosis through several layers creates a consistent mechanistic framework between chromatin regulation and cell death execution (Pandeya and Kanneganti, 2024[56]; Tweedell et al., 2024[68]). Epigenetic silencing of chemoresistant cancers is caused by DNA methylation of PANoptotic effectors (RIPK3, GSDME, CASP8) (Martinez et al., 2007[50]). Transcriptional permissiveness to death gene induction is determined by histone acetylation of PANoptotic enhancers and chromatin accessibility by SWI/SNF and NuRD complexes (Baxter et al., 2023[4]; Wolf et al., 2024[79]). Regulatory domains are determined by the histone methylation status of H3K27me3 repression and H3K9me3 protection (Zheng and Kanneganti, 2020[86]). Non-coding RNA networks form post-transcriptional regulatory nodes: miR-223-3p convergently inhibits pyroptosis and necroptosis, lncRNA NEAT1 inflammasome scaffolding, and circRNA inhibitory miRNA sequestration. These three levels of epigenetics are incorporated into measurable parameters of PANoptotic competence (Pandeya and Kanneganti, 2024[56]). Increasing discrete epigenetic phenotypes by multiplying specific PANoptosome structural components is extraordinarily novel, and the multiplication of epigenetic status to measurable cell death competence parameters. System-level interactions between the methylome, chromatin dynamics, and ncRNA regulation produce quantitative epigenetic thresholds that define PANoptotic sensitivity and disease progression.

**Figure 2:**
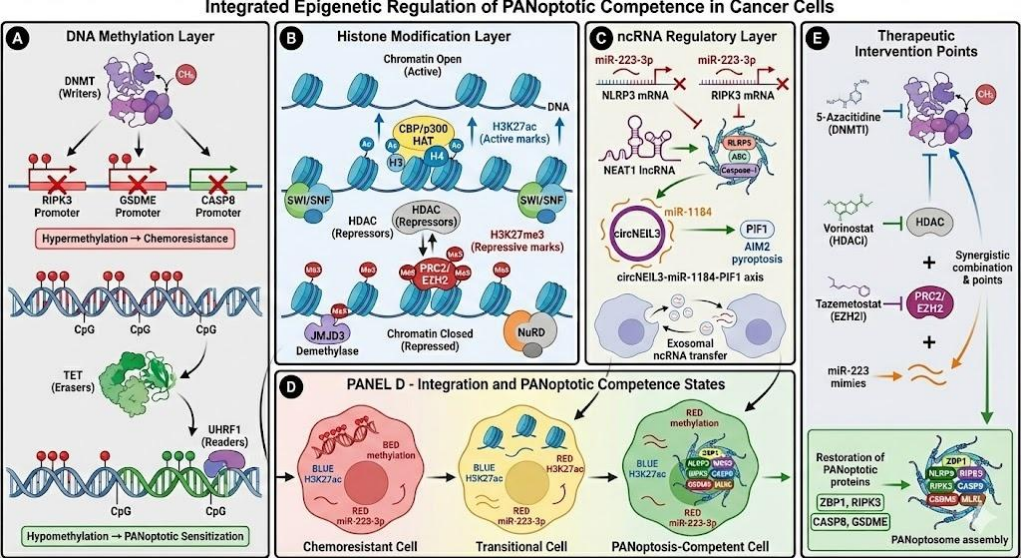
Epigenetic control of PANoptotic competence: multi-layer regulation and therapeutic intervention

Figure 2(Fig. 2) depicts that three epigenetic layers, namely DNA methylation (Panel A), histone modifications (Panel B), and ncRNA networks (Panel C), work together to form quantifiable competence states of PANoptotic. Such integration defines the cellular phenotypes between chemoresistant cells characterized by high methylation and inhibited PANoptotic genes (Panel D, red) and PANoptosis-competent cells characterized by low methylation and re-expression of effectors (Panel D, green). Discrete epigenetic checkpoint (Panel E) therapeutic intervention restores PANoptotic competency and surpasses tumor immunoresistance.

### Biomarker-guided precision oncology strategy

The stratification of epigenetics allows for patient selection and therapy optimization (Zhu et al., 2024[90]). Methylation signatures of chemoresistance and immunotherapy response are high-resolution CpG methylation profiles using PANoptotic component loci (RIPK3, GSDME, CASP8, ZBP1, NLRP3, and STING) (Ohashi et al., 2019[54]). At the same time, transcriptional competence landscapes are shown by histone modification mapping (H3K27ac, H3K27me3, H3K9me3) (Zheng and Kanneganti, 2020[86]), whereas post-transcriptional regulatory status is shown by ncRNA panel analysis (miR-223-3p dosage, NEAT1 abundance, circNEIL3 levels). These three epigenetic datasets were combined to form detailed PANoptotic competence profiles. Highly silenced PANoptotic effectors Patients whose cancer cells are highly methylated should be considered for epigenetic priming with DNA methyltransferase inhibitors (DNMTi: 5-azacitidine, decitabine, guadecitabine) followed by conventional chemotherapy or PANoptosis agonists. Histone deacetylase inhibitors (HDACi) and bromodomain inhibitors are useful in patients with HDAC-mediated repression (Chiappinelli et al., 2015[10]; Roulois et al., 2015[61]). Individuals with ncRNA patterns out of control will need either ncRNA replacement (miR-223 mimics) or lncRNA destabilization (NEAT1 inhibitors). This targetable modularity of PANoptosis can be used to turn PANoptosis into a more effective mechanistic concept as well as precision medicine that can be used to select therapeutic choices in an individualized fashion in immunoresistant tumors (Ros et al., 2025[60]) (Table 2(Tab. 2); References in Table 2: Arroyo Villora et al., 2024[1]; Bennett et al., 2023[5]; Deng and Hu, 2023[15]; Huang et al., 2020[26]; Kim et al., 2023[35]; Konno et al., 2018[37]; Liu et al., 2024[46]; Long et al., 2020[48]; Martinez et al., 2007[50]; Tan et al., 2021[67]; Zhang et al., 2022[84]; Zhou et al., 2024[88]).

**Figure 3:**
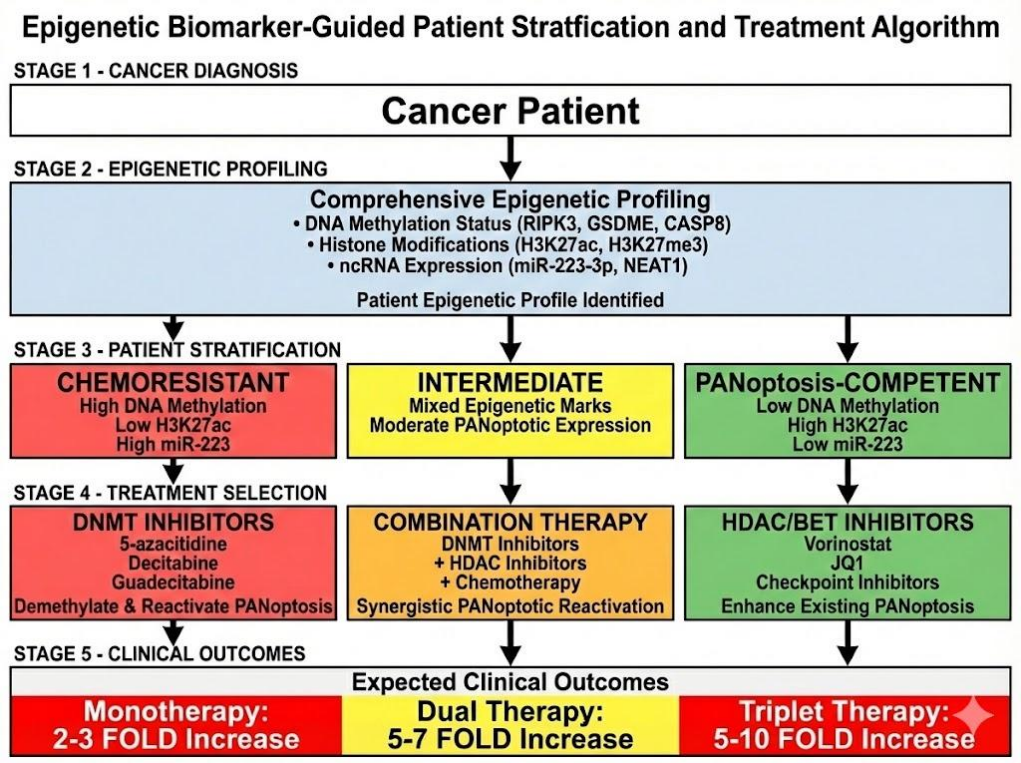
Patient stratification and epigenetic biomarker-guided treatment strategy for PANoptosis reactivation

Epigenetic stratification makes it possible to select patients and optimize therapy. CpG methylation signatures of chemoresistance and immunotherapy response are high-resolution CpG methylation profiles of PANoptotic component loci (RIPK3, GSDME, CASP8, ZBP1, NLRP3, and STING). Transcriptional competence topographies are depicted by histone modification mapping (H3K27ac, H3K27me3, and H3K9me3), and post-transcriptional regulatory status is determined by ncRNA panel analysis (miR-223-3p dosage, NEAT1 abundance, and circNEIL3 levels). The combination of these three epigenetic datasets resulted in detailed PANoptotic competence profiles (Figure 3(Fig. 3)).

### Combination therapeutic strategies

PANoptosis can be rationally targeted by epigenetic combinations (Chiappinelli et al., 2015[10]; Roulois et al., 2015[61]). The combination of DNMT and HDAC inhibition enhances endogenous retroviral element-derived dsRNA, resulting in improved interferon responses and immunotherapy. 5-azacitidine + HDACi combinations show enhanced epigenetic reprogramming of PANoptotic loci (Gong et al., 2023[20]). Anti-HER2 therapy (trastuzumab) of DNMT/HDAC priming increases ZBP1-induced immunogenic cell death. BET bromodomain and TNF-α signaling modulator combinations can be exploited to optimize PANoptotic execution (Wang et al., 2017[76]), whereas miR-223 replacement coupled with NEAT1 inhibition can be used as ncRNA-guided combinations to provide enhanced post-transcriptional control (Karki et al., 2021[33]). Epigenetic regulation is organized in a manner that allows customized combinations of epigenetic regulation to meet the requirements of individual patient epigenetic profiles, thereby enhancing the therapeutic effect and reducing off-target immunopathology.

## Future Research Directions and Clinical Translation

There are critical knowledge gaps that need to be addressed through urgent research. The mapping of locus-specific methylation of the entire PANoptotic pathway (ZBP1 -ASC-CASP1-CASP8-RIPK3-MLKL-GSDMD/GSDME) is lacking; high-resolution CpG profiling is needed. High-resolution imaging studies are necessary to understand the spatiotemporal control of PANoptosome assembly and how endosomal trafficking and mitochondrial functioning are coordinated using epigenetic priming at membrane microdomains. The role of epitranscriptomic modifications (m6A, pseudouridine) in the regulation of PANoptosis has not been investigated. Clinical trials are needed to evaluate prospective randomized trials of epigenetic-based biomarker stratification of DNMT/HDAC/BET inhibitors as PANoptosis sensitizers. High-resolution methylation profiling and ncRNA quantification should be implemented in clinical laboratories in real-life scenarios. Such combinations of epigenetic priming with traditional cytotoxic and immunotherapy reagents require optimization according to tumor type and patient group. Notably, the mechanisms by which immunopathology and tissue toxicity result in overactivity of PANoptosis can be inhibited in a systematic way, especially in the context of sterile inflammation.

## Conclusion

The epigenetic regulation of PANoptosis allows it to be a measurable and therapeutically adjustable program of immunogenic cell death in cancer. Combining non-coding RNA networks, histone modifications, and DNA methylation into integrated parameters of epigenetic competence allows oncology approaches to be precise in tumors that were previously immunoresistant. Epigenetic status patient stratification will revolutionize cancer immunotherapy, whereby it selects the best combination of therapeutic options and anticipates the response. Clinical success in the future will depend on the systematic characterization of PANoptotic epigenetic landscapes, the development of selective epigenetic modulators, and effective clinical trials to prove the concept of epigenetic-directed PANoptosis activation. This detailed mechanistic insight makes epigenetic PANoptosis biology a strategic base upon which personalized cancer immunotherapy can be advanced and incurable malignancies can be enhanced.

## Declaration

### Ethics approval and consent to participate 

Not applicable. 

### Consent for publication 

Not applicable. 

### Availability of data and materials 

Not applicable. 

### Competing interest 

The authors declare that they have no competing interest. 

### Funding 

The authors extend their appreciation to the Deanship of Research and Graduate Studies at King Khalid University for funding this work through Large Group Project under grant number RGP.2/630/46.

### Artificial Intelligence (AI) - Assisted Technology

The authors declare that AI was not used for the preparation of this manuscript. 

### Author's contribution 

Yogendra Singh, Muhammad Afzal: Investigation, Writing-original draft & Formal analysis; M Arockia Babu, Surya Nath Pandey, A Rekha: Visualization; Gaurav Gupta, Imran Kazmi: Formal analysis; Sami I Alzarea, Omar Awad Alsaidan: Resources, Investigation & Conceptualization; Waleed Hassan Almalki, Salem Salman Almujri: Supervision, Investigation & Conceptualization. 

## Figures and Tables

**Table 1:**
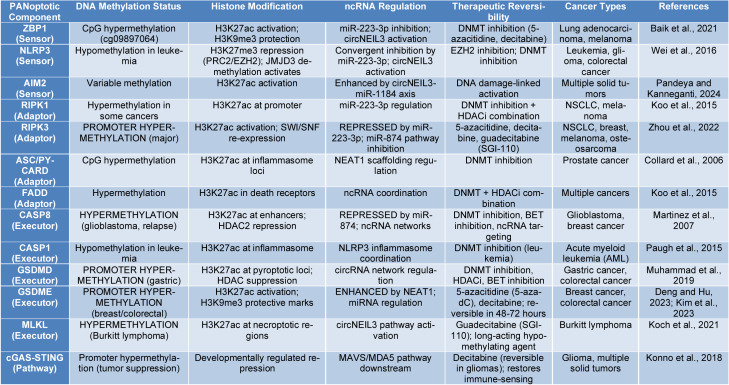
Epigenetic regulation of panoptotic components and therapeutic reversibility in cancer

**Table 2:**
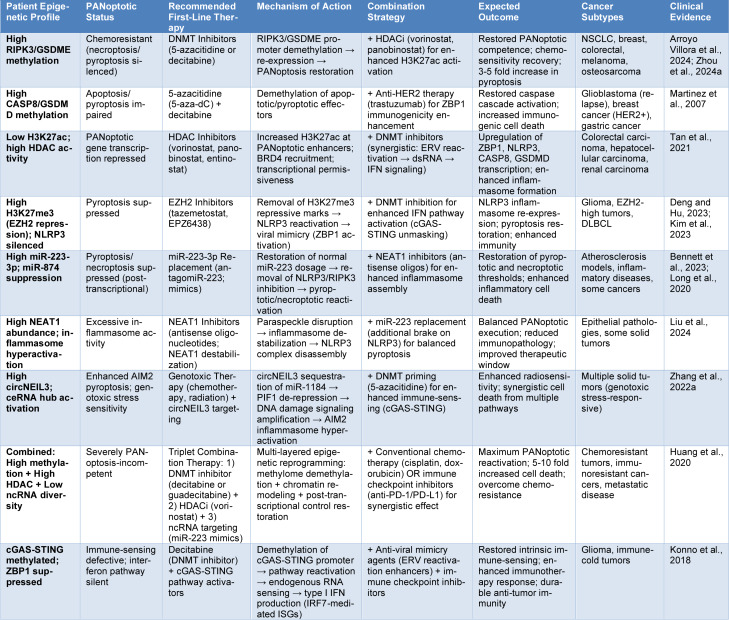
Multi-targeted epigenetic biomarker-guided treatment strategies for panoptosis-based cancer immunotherapy

**Figure 1:**
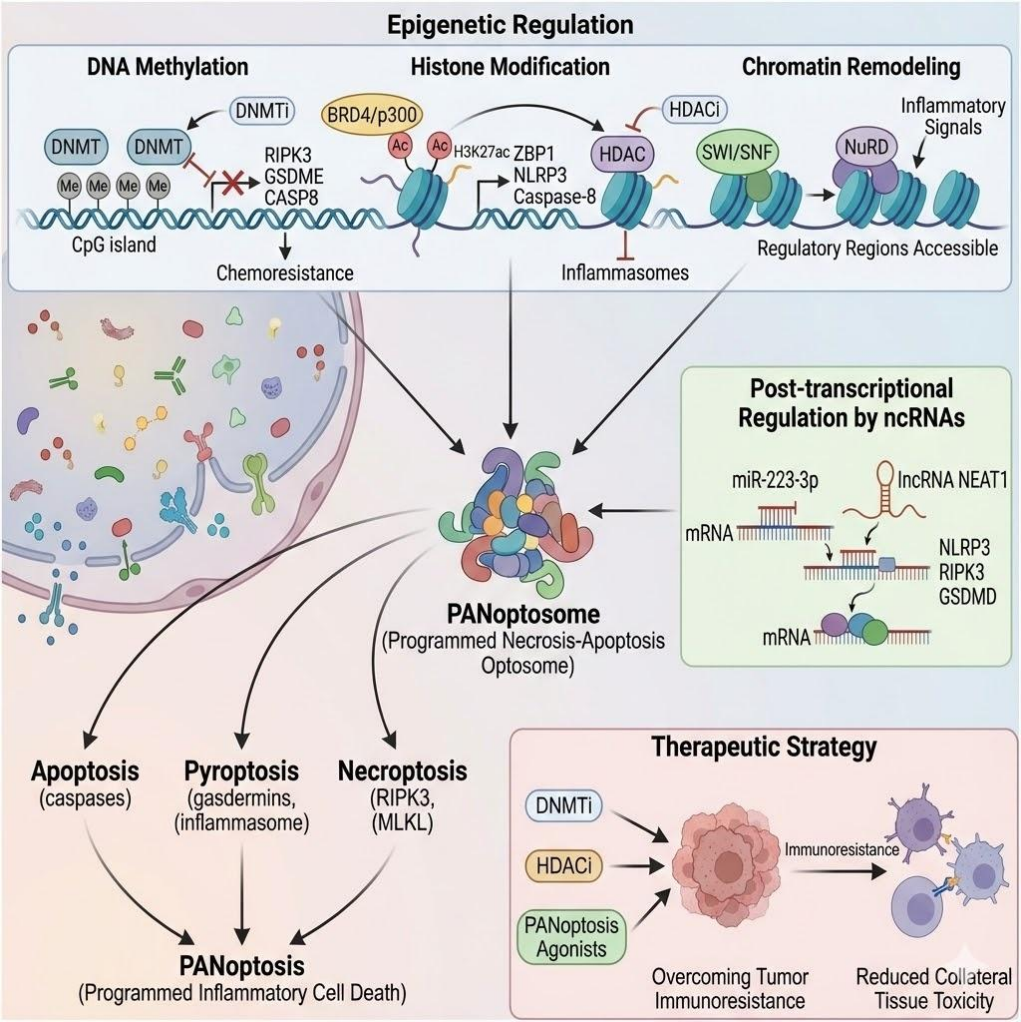
Graphical abstract
